# Study on the relationship between fixation characteristics and hit rate in psychological procedure training of free throw

**DOI:** 10.1371/journal.pone.0293436

**Published:** 2024-05-09

**Authors:** Chunzhou Zhao, Na Liu, Sunnan Li

**Affiliations:** 1 Beijing Normal University-College of P.E and Sports, Beijing, China; 2 Guangdong Country Garden Polytechnic-School office, Qingyuan, China; University of Castilla-La Mancha, SPAIN

## Abstract

**Background:**

Free throw is an important means of scoring in basketball games. With the improvement of basketball competition level and the enhancement of confrontation degree, the number of free throws in the game gradually increases, so the score of free throw will have an important impact on the result of the game. The purpose of this study is to explore the relationship between visual attention characteristics and hit rate of basketball players in free throw psychological procedure training, so as to provide scientific basis for basketball teaching and training.

**Methods:**

Forty players with similar free throw abilities were randomly assigned to the experimental group (10 males, 10 females) and control group (10 males, 10 females). The experimental group was free throw psychological procedure training, while the control group was trained with routine training, Eye movement indices (number of fixations, fixation duration, and pupil dilation) and the free throw hit rate and analyzed before and after the experiment. Group differences were examined using t-tests, while paired sample t-tests were conducted to compare pre- and post-test results within each group. The training time and training times of the two groups were the same.

**Results:**

There were significant differences in fixation duration, number of fixations, pupil diameter and free throw hit rate between pre-test and post-test in the experimental group (*P* < 0.05). Post-test, there were significant differences in number of fixations, fixation duration, pupil diameter and free throw hit rate between the two groups (*P* < 0.05). There was a significant positive correlation between number of fixations and free throw hit rate in top (*P* < 0.01), and there was a significant positive correlation between fixation duration and hit rate in front (*P* < 0.01).

**Conclusions:**

The psychological procedure training can improve the visual information search strategy and information processing ability of free throw, and significantly improve the free throw hit rate. There was a positive correlation between the front fixation time and the free throw hit rate, and there was a positive correlation between the top number of fixations and the free throw hit rate.

## Introduction

With the improvement of the level of basketball competition, the competition is more intense, and the number of free throws is gradually increasing. In modern professional league games, there are about 50 free throws per game, and in some games, the free throw hit rate determines the win or loss of the game [1]. The free throw technique is the only closed skill in basketball [2], and the stability of the movement is very important to the closed skill. Nideffer believes that in competition, closed skills require a high degree of attention control to avoid interference from external distractors [3]. The free throw is a complex targeting skill that requires the integration of visual information gained through overt shifts of gaze with effector movements that carry out the aiming movement [4]. Shooting quality is mainly determined by the mechanics of shooting at the basket, the positions of the eyes (due to the fact that looking at the target has an important role in improving shooting precision) and head, and concentration (due to the fact that it is an important role in optimizing shooting precision) [5].

Visual attention is very important in basketball shooting, which is a prerequisite for the formation of good muscle proprioception and the improvement of the hit rate [6]. Cognitive resource theory considers attention as a cognitive resource for the recognition and processing of stimulus information. Its capacity and energy are limited, and the allocation mechanism of cognitive resources is flexible. It can be adjusted and controlled according to people’s actual needs, and give priority to processing tasks that it thinks are more important [7]. When making a free throw, it is impossible to search and process all the information equally, but only selective search and processing of the information, so as to ensure the fine processing of the information that has an important impact on the shooting rate of the free throw. Experienced players detect key visual information that may predict successful shooting by appropriately moving their gaze based on the shooter’s movement [8]. When the line of sight was obscured before the start of the shooting movement, the performance decreased significantly.

The human brain has plasticity, and training specific areas of the human brain through scientific methods and means can improve its function and develop the potential of the brain [9,10]. One important factor that has been shown to help better understand basket-ball shooting accuracy is visual attention [5]. Visual attention training can also alter the mechanism of executing free throw. Vickers, et al. through the implementation of static eye training on college students’ shooting, found that the shooting rate of the static eye training group was significantly higher than that of the technical training group, and believed that the neural network organization of task control was optimized by the static eye training [11]. Du & Wu intervened the position of aiming basket points in shooting training, and the shooting rate of participants was significantly improved after 9 weeks [12]. Jimene et al. used 6 weeks of on-site training plus visual-perceptual training intervention, and the results showed that the on-site performance of athletes was significantly improved [13]. Some scholars also believe that before the free throw, players’ stable mood and relatively continuous concentration will promote their athletic performance [14,15]. Harle and Vickers conducted quiet eye training on college female basketball players, and the results proved that quiet eye training could significantly improve the athletes’ free throw hit rate [16].

Free throw hit rate is a commonly used indicator to evaluate the efficiency of free throw [17]. The free throw hit rate is closely related to many factors, and the choice of fixation position during the free throw is crucial as long as [18]. Once attention is stabilized on the target, information is processed and processed [19]. Numerous experiments have shown that human visual attention ability can be improved and strengthened by training [20]. At the same time, it also shows that in the scientific training of basketball, the role and value of sports psychological training in improving the level of basketball competition has been highly concerned and universally recognized by professionals. Especially in recent years, the research on improving the shooting rate of basketball players through psychological intervention has also shown an upward trend. The free throw has not only a complete action procedure but also a stable psychological procedure. In teaching or training, most teachers or coaches often only pay attention to the action procedure and the standardization of the action, but ignore the guidance of the psychological procedure training. The first hypothesis of this study is that the psychological procedure training can optimize the visual attention of players during free throw and improve the free throw hit rate. The second hypothesis is that there is a positive relationship between the fixation characteristics of the players during the free throw and the free throw hit rate. In view of this, this study adopts the tracking experimental design and adopts the free throw psychological procedure training of Kevin et al., to explore the influence of the 8-week free throw psychological procedure training on the visual characteristics and free throw hit rate of players, so as to provide guidance for basketball teaching and training in the future.

## Materials & methods

### Participants

This research used G*Power 3.1.9.7 (Germany) software to estimate the sample size and set the effect size of 0.03 and two-tailedα = 0.05 [21], and a statistical power of 0.80 was reached for 40 participants [22]. 40 participants were divided into experimental groups (n = 20, 10 women, 10 men) and control groups (n = 20, 10 women, 10 men) according to their gender and free throw hit rate. All participants were from the men’s and women’s basketball teams of Beijing Normal University, and they all participated in the highest level basketball league of Chinese University students. The age range of participants was 18–22 years (mean:20.56.; SD:2.47 years), with more than 10 years of experience per participants (mean:10.09; SD: 1.96 years) and over 15 hours (mean:15.02; SD: 2.86 hours) of training per week in the past year. All participants participated in the experiment and received compensation for their time. The study was approved by the Ethics Committee of Beijing Normal University-College of P.E and Sports (No. 20221126). All of the participants provided written informed consent prior to the start of the experiment, they were right-handed, with normal or corrected vision. Before the experiment, the eye movement characteristics and free throw hit rate of the experimental group and the control group were at the same level (*P* > 0.05).

### Apparatus

The experiment used the Tobii Glasses 3 portable eye tracker produced in Sweden, with a sampling rate of 100Hz. This instrument does not cause any obstruction to the wearer’s field of view and provides maximum freedom of head and body movement without compromising data quality. We make sure to capture nature and authentic behavior to the greatest extent possible.

### Design

The experiment was an intersubject design. The independent variables were the experimental group and the control group, and the dependent variables were eye movement indexes (number of fixations, fixation duration, pupil dilation) and free throw hit rate.

### Procedure

The experimental procedure consisted of three stages: pre-test, mid-test, and post-test. The experimentation was conducted at the basketball stadium of Beijing Normal University from March 2, 2023 to April 30. The pre-test period is scheduled for March 2–3, 2023. eye movement data test, the experimental group and the control group wore an eye tracker to free throw, and each participant recorded eye movement data three times, the whole experiment took about 150 minutes. The free throw hit rate test was arranged in the morning and afternoon of each day. Each person made 5 free throws as a group, and the interval was 3 minutes. Two groups were tested in the morning and afternoon, and the test was conducted for 5 consecutive days, a total of 100 times were tested, and the number of successful and failed free throws was recorded. The mid-test period is scheduled for April 1–2, 2023; the post-test period is scheduled for April 29–30, 2023. The same methodology was employed for both the mid-test and post-test as in the pre-test phase. Psychological procedure training will be conducted from March 6 to April 28.

## Experimental group training methods

The psychological procedure of Kevin, et al. was used as the training method for the experimental group. The specific procedures were as follows: (1) standing at the free throw line quickly imagining or thinking of "basket" or "hole"; (2) thinking "relax" or "calm"; (3) count the number of dribbles; (4) thinking "relax" or "calm"; (5) thinking "basket" or "hole"; (6) thinking about "random movements" or "hand shapes" [23]. Prior to implementing the psychological procedure training for free throw, a comprehensive explanation of the content, requirements, and precautions associated with this training was provided to all athletes in the experimental group. Once all athletes fully comprehended and acquired proficiency in the psychological procedure for free throw, they commenced its implementation during their free throw training sessions. During the implementation process, all athletes in the experimental group were required to strictly adhere to the prescribed psychological procedures for each free throw practice.

### Control group training methods

The control group underwent training using the conventional free throw method, where two individuals in a group would alternate between performing free throws and assisting each other in retrieving the ball, while also providing corrective feedback on their respective shooting techniques. The instructor supervised the entire training session. The only distinction between the control group and the experimental group was that no mental program for free throw training was utilized.

### Dose of training

The training lasted for 8 weeks, 3 times a week, 60 minutes each class, 200 times each class, and the total number of free throws was 4800 times.

### Experimental control

The psychological procedure of free throw training was used in the experimental group, and the conventional free throw training was used in the control group. The free throw training time and times were the same in the two groups. The physical education courses of the two groups in the school are completely the same, and the same basketball teacher teaches. Besides normal extracurricular activities, there is no extra practice in the experimental class and the control class.

### Division of area of interest (AOI)

**Fig 1:**
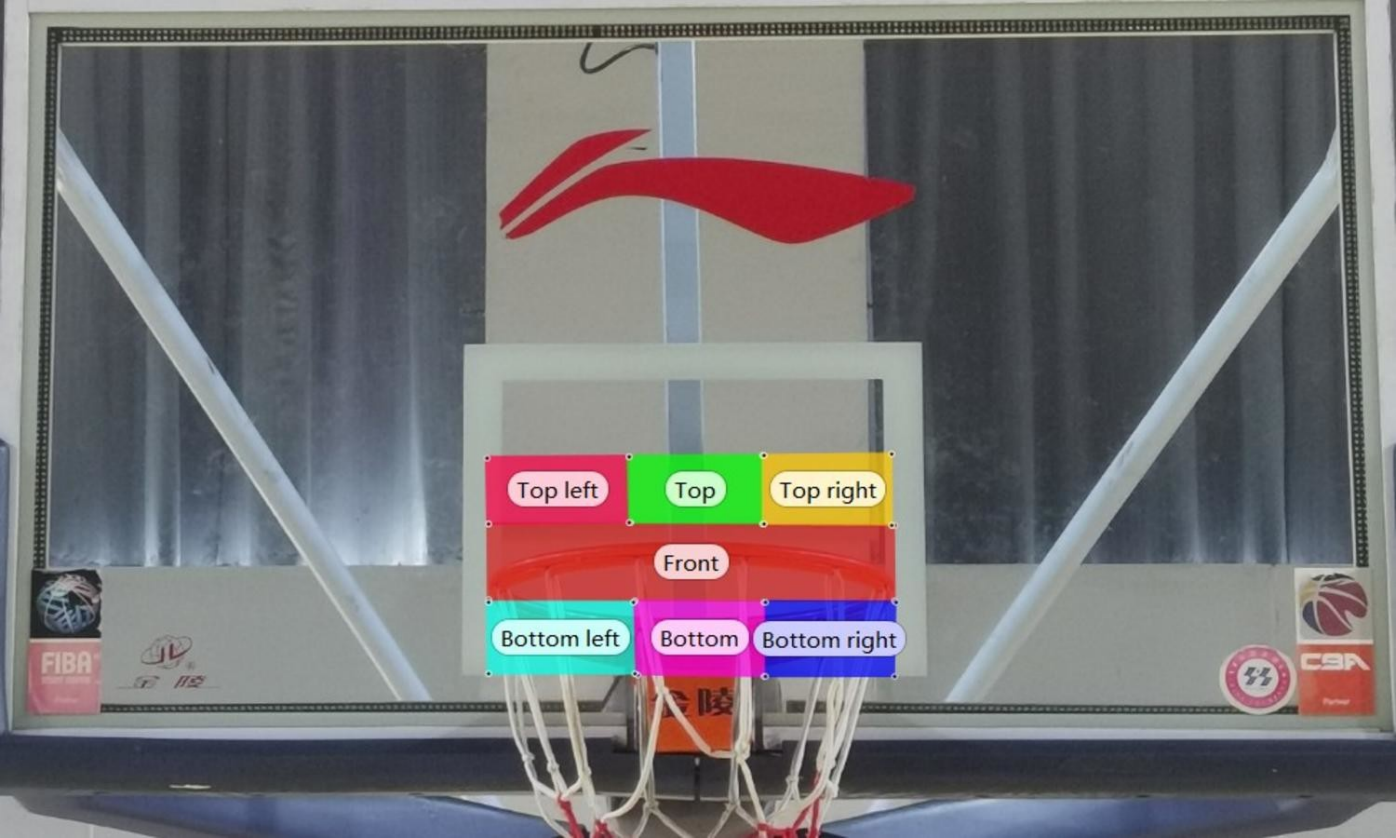
AOI based on free-throw fixation position.

Area of Interest (AOI) is the part of the visual field that the subject focuses on during information search. After asking more basketball experts, scholars and instrument engineers, in this experiment, AOI were divided into seven regions based on the locations that the subjects fixated on during the free-throw shooting process. (See Fig 1)

### Data analysis

The data were subjected to analysis using a two-way repeated measures analysis of variance (ANOVA). The homogeneity of variance was assessed by employing the Mauchly sphericity test, and adjustments were made using the Greenhouse-Geisser method when violations of sphericity assumptions occurred. Post hoc tests were conducted utilizing the least significant difference (LSD) procedure.

### Ethics statement

This study was conducted in accordance with the recommendations of the Ethics Committee of the College of Physical Education and Exercise, Beijing Normal University, and written informed consent was obtained from all participants. Participants gave written informed consent in accordance with the Declaration of Helsinki. The experimental protocol was approved by the Ethics Committee of Beijing Normal University (No. 20221126).

## Results

### Number of fixations

After S-W test, it was observed that the data from each group exhibited a normal distribution. The Mauchly sphericity test was conducted on the front, top, bottom, top left, bottom right, top right and bottom right positions. The results revealed that Machly W values were 0.51, 0.494, 0.231, 0.290, 0.301, 0.266 and 0.110 respectively (*P* < 0.001), indicating non-conformity to the required level of sphericity for inspection criteria according to Moche’s standards of assessment degree of conformity to spherical shape requirements in this study design. Therefore, corrected results using one-way ANOVA ’Greenhouse-Geisser’ will be employed.

The results of the repeated measures ANOVA are presented in Table 1. The main effect of group was not found to be statistically significant for any of the areas: front (F = 0.436, p = 0.513, η^2^ = 0.011), top (F = 0.284, p = 0.597, η^2^ = 0.007), bottom (F = 2.978, p = 0.093, η^2^ = 0.073), top left (F = 0.109, p = 0.743, η^2^ = 0.003), bottom left (F = 1.032, p = 0.316, η^2^ = 0.026), top right (F = 0.011, p = 0.917, η^2^ = 0.000), and bottom right (F = 0.676, p = 0.416, η^2^ = 0.017). The number of measurements had a significant main effect in the following areas: top left (F = 4.085, p = 0.025, η^2^ = 0.181), bottom (F = 5.891, p = 0.010, η^2^ = 0.134), bottom left (F = 8.027, p = 0.005, η^2^ = 0.174), and top right (F = 4.104, p = 0.044, η^2^ = 0.097). However, there was no statistically significant main effect of the number of measurements in the following areas: front (F = 2.062, p = 0.152, η^2^ = 0.051), top (F = 0.840, p = 0.395, η^2^ = 0.022), and bottom right (F = 0.117, p = 0.749, η^2^ = 0.003). The interaction effect of the number of measurements per group demonstrated statistical significance within the following areas: front (F = 6.742, p = 0.007, η^2^ = 0.151), top (F = 5.249, p = 0.018, η^2^ = 0.121), bottom (F = 7.776, p = 0.003, η^2^ = 0.170), bottom right (F = 7.140, p = 0.010, η^2^ = 0.158) and top left (F = 1.011, p = 0.374, η^2^ = 0.052). However, no statistically significant interaction effect was observed between the number of measurements and group in the bottom left (F = 0.034, p = 0.890, η^2^ = 0.001) and in the top right (F = 2.335, p = 0.130, η^2^ = 0.058).

**Table 1:** Comparison of the number of fixations for each OAI between the experimental and control groups pre- and post-experiment (unit: Time).

	1	F/P	front	top	bottom	top left	bottom left	top right	bottom right
experimental group	pre-test		2.15±0.67	2.00±0.79	3.35±0.93	1.25±0.55	1.60±0.88	1.15±0.99	0.80±0.52
	mid-test		2.05±0.51	1.90±0.72	3.00±0.92	1.20±0.52	1.50±0.83	1.00±0.76	0.75±0.55
	post-test		2.25±0.79	2.25±0.72	2.25±0.72	1.35±0.59	1.15±0.88	0.50±0.51	0.40±0.50
control group	pre-test		2.50±1.15	2.05±0.89	3.30±1.08	1.20±0.52	1.80±0.77	0.95±0.76	0.70±0.86
	mid-test		2.40±1.05	1.95±0.76	3.20±1.01	1.20±0.52	1.70±0.66	0.90±0.64	0.70±0.84
	post-test		2.05±0.83	1.80±0.70	3.35±0.99	1.60±0.75	1.40±0.68	0.85±0.49	1.00±0.73
repeated measures F test	group main effect	F	0.436	0.284	2.987	0.109	1.032	0.011	0.676
		P	0.513	0.597	0.093	0.743	0.316	0.917	0.416
	number of tests main effect	F	2.062	0.840	5.891	6.544	8.027	4.104	0.117
		P	0.152	0.395	0.010	0.011	0.005	0.44	0.749
	group * Number of tests	F	6.742	5.249	7.766	1.900	0.034	2.335	7.140
		P	0.007	0.018	0.003	0.174	0.897	0.058	0.010

The results of the group simple effect test indicated that in the pre-test, the simple effect of group was not statistically significant: front (F = 1.387, p = 0.246, η^2^ = 0.035), top (F = 0.035, p = 0.852, η^2^ = 0.001), bottom (F = 0.025, p = 0.876, η^2^ = 0.001), top left (F = 0.087, p = 0.770, η^2^ = 0.002), bottom left (F = 0.585, p = 0.449, η^2^ = 0.015), top right (F = 0.515, p = 0.477,η^2^ = 0.013) and bottom right(F = 0.196, p = 0.661, η^2^ = 0.005). The mid-test revealed no significant simple effect of group: front (F = 1.808, p = 0.187, η^2^ = 0.045), top (F = 0.046, p = 0.832, η^2^ = 0.001), bottom (F = 0.432, p = 0.515, η^2^ = 0.011), top left (F = 0.432, p = 0.515, η^2^ = 0.011), bottom left (F = 0.097, p = 0.757, η^2^ = 0.003), top right (F = 0.717, p = 0.402, η^2^ = 0.019) and bottom right(F = 0.048, p = 0.187, η^2^ = 0.045). The simple effect of group was not statistically significant in the post-test for front (F = 0.615, p = 0.438, η^2^ = 0.016), top (F = 4.061, p = 0.051, η^2^ = 0.097), top left (F = 1.369, p = 0.249, η^2^ = 0.035), and bottom left (F = 1.017, p = 0.320, η^2^ = 0.026). However, a statistically significant simple effect of group was observed for bottom (F = 16.247, P < 0.001, η^2^ = 0.300), top right (F = 4.874, p = 0.033, η^2^ = 0.114); and bottom right (F = 9.243, p = 0.004, η^2^ = 0.196).

In the psychological procedure of free throw training, the number of measurements had a significant simple effect in the following areas: top (F = 3.769, p = 0.032, η^2^ = 0.169), bottom (F = 9.154, p = 0.001, η^2^ = 0.331), top right (F = 3.385, p = 0.045, η^2^ = 0.155), and bottom right (F = 3.847, p = 0.030, η^2^ = 0.172). However, there was no statistically significant difference observed in the simple effect of number of measurements across these areas: front (F = 2.018, p = 0.147, η^2^ = 0 .098), top left (F = 0.775, p = 0.468, η^2^ = 0.040), and bottom (F = 2.573, p = 0.090, η^2^ = 0.122). The number of measurements had a significant simple effect on conventional training: front (F = 4.498, p = 0.018, η^2^ = 0.195), top left, (F = 4.332, p = 0.021, η^2^ = 0.189). However, the simple effect of the number of measurements was not significant for top (F = 1.723, p = 0.194, η^2^ = 0.085), bottom (F = 0.407, p = 0.669, η^2^ = 0.022), bottom left (F = 2.105, P = 0.136, η^2^ = 0.102), top right (F = 0.169, P = 0.845, η^2^ = 0.009) and bottom right conditions (F = 1.459, P = 0.246, η^2^ = 0.073).

The results of multiple comparisons revealed significant differences in fixation duration during the psychological procedure of free throw training across various areas: top (post-test and mid-test, p = 0.018); bottom (pre-test, post-test, and mid-test, p < 0.001; p = 0.015); bottom left (post-test and pre-test, p = 0.029); top right (post-test, pre-test, and mid-test, p = 0.012, p = 0.020); bottom right (post-test and pre-test, p = 0.027). No significant differences were observed in other areas (p > 0.05).

The number of fixations exhibited significant differences across various areas with conventional training: front (pre-test and mid-test, post-test; p = 0.004, p = 0.013), top left (pre-test and post-test, p = 0.010) as well as (mid-test and post-test, p = 0.005), and bottom left (pre-test and post-test p = 0.029). No significant differences were observed in other areas (p > 0.05).

### Fixation duration

After S-W test, it was observed that the data from each group exhibited a normal distribution. The Mauchly sphericity test was conducted on the front, top, bottom, top left, bottom right, top right and bottom right positions. The results revealed that Machly W values were 0.263, 0.010, 0.019, 0.079, 0.003, 0.001 and 0.002 respectively (p < 0.001), indicating non-conformity to the required level of sphericity for inspection criteria according to Moche’s standards of assessment degree of conformity to spherical shape requirements in this study design. Therefore, corrected results using one-way ANOVA ’Greenhouse-Geisser’ will be employed.

The results of the repeated measures ANOVA are presented in Table 2. The main effect of group was not found to be statistically significant for any of the areas: front (F = 0.004, p = 0.949, η^2^ = 0.000), top (F = 0.176, p = 0.677, η^2^ = 0.005), bottom (F = 0.278, p = 0.601, η^2^ = 0.007), top left (F = 0.314, p = 0.579, η^2^ = 0.008), bottom left (F = 0.007, p = 0.936, η^2^ = 0.000), top right (F = 0.228, p = 0.635, η0.006), and bottom right (F = 0.307, p = 0.583, η^2^ = 0.008).

**Table 2:** Comparison of the fixation duration for each OAI between the experimental and control groups pre- and post-experiment (unit: Ms).

		F/P	front	top	bottom	top left	bottom left	top right	bottom right
experimental group	pre-test		410±87	369±75	305±96	327±0.92	300±61	206±142	159±121
	mid-test		414±78	372±73	304±97	322±0.49	299±41	204±141	158±420
	post-test		553±70	480±75	342±77	335±60	386±77	185±133	63±98
control group	pre-test		442±116	386±76	323±59	329±49	319±41	203±123	133±137
	mid-test		445±99	386±76	322±58	328±48	319±41	203±123	131±135
	post-test		486±116	425±91	338±51	351±42	344±39	228±136	157±148
repeated measures F test	group main effect	*F*	0.004	0.176	0.278	0.314	0.007	0.228	0.307
		*P*	0.949	0.677	0.601	0.579	0.936	0.635	0.583
	number of tests main effect	*F*	20.225	18.819	4.342	1.568	14.179	0.008	1.135
		*P*	<0.001	<0.001	0.044	0.219	0.001	0.928	0.294
	group * Number of tests	*F*	5.704	4.260	0.727	0.230	0.4.456	0.447	3.216
		*P*	0.018	0.046	0.400	0.644	0.041	0.508	0.081

The number of measurements had a significant impact on the results, as indicated by statistical analysis: front (F = 20.225, p < 0.001, η^2^ = 0.347); top (F = 18.819, p<0.001, η^2^ = 0.331), bottom (F = 4.342, p = 0.044, η^2^ = 0.103), bottom left (F = 14.179, p = 0.001, η^2^ = 0.272). However, there was no statistically significant main effect of the number of measurements in the following areas: top (F = 0.840, p = 0.395, η^2^ = 0.022), top left (F = 1.568, p = 0.219, η^2^ = 0.040), top right (F = 0.008, p = 0.928, η^2^ = 0.000), bottom right(F = 1.135, p = 0.294, η^2^ = 0.029). The interaction effect of the number of measurements per group demonstrated statistical significance within the following areas: front (F = 5.704, p = 0.018, η^2^ = 0.131), top (F = 4.260, p = 0.044, η^2^ = 0.101), bottom left (F = 4.456, p = 0.041, η^2^ = 0.105).However, no statistically significant interaction effect was observed between the number of measurements and group in the bottom (F = 0.727, p = 0.400, η^2^ = 0.019), top left (F = 0.230, p = 0.644, η^2^ = 0.006),top right (F = 0.447, p = 0.508, η^2^ = 0.012), bottom right(F = 3.216, p = 0.081, η^2^ = 0.078).

The results of the group simple effect test indicated that in the pre-test, the simple effect of group was not statistically significant: front (F = 0.948, p = 0.336, η^2^ = 0.024), top (F = 0.499, p = 0.484, η^2^ = 0.013), bottom (F = 0.511, p = 0.479, η^2^ = 0.013), top left (F = 0.012, p = 0.915, η^2^ = 0.000), bottom left (F = 1.399, p = 0.244, η^2^ = 0.036), top right (F = 0.002, p = 0.962, η^2^ = 0.000) and bottom right(F = 0.404, p = 0.529, η^2^ = 0.011). The mid-test revealed no significant simple effect of group: front (F = 1.167, p = 0.287, η^2^ = 0.030), top (F = 0.387, p = 0.542, η^2^ = 0.010), bottom (F = 0.511, p = 0.479, η^2^ = 0.013), top left (F = 0.106, p = 0.746, η^2^ = 0.003), bottom left (F = 1.600,p = 0.214, η^2^ = 0.040), top right (F = 0.717, p = 0.402, η^2^ = 0.019) and bottom right(F = 0.430, p = 0.516, η^2^ = 0.011). The simple effect of group was not statistically significant in the post-test for top left (F = 1.079, p = 0.305, η^2^ = 0.028), bottom left (F = 4.884, p = 0.033, η^2^ = 0.114), bottom (F = 0.029, p = 0.866, η^2^ = 0.001), top right (F = 1.017, p = 0.320, η^2^ = 0.026). However, a statistically significant simple effect of group was observed for front (F = 4.817, p = 0.034, η^2^ = 0.113), top (F = 4.349, p = 0.044, η^2^ = 0.103); and bottom right (F = 5.610, p = 0.023, η^2^ = 0.129).

The psychological procedure of free throw training, the simple effect of the number of measurements was significant in the following areas: front (F = 13.663, p < 0.001, η^2^ = 0.425), top (F = 10.354, p < 001, η^2^ = 0.359), bottom (F = 9.154, p = 0.001, η^2^ = 0.331), bottom left (F = 10.213, p < 0.001, η^2^ = 0.356), bottom right (F = 2.117, p = 0.135, η^2^ = 0.103). However, no significant effects were observed in the bottom (F = 12.269, p = 0.118, η^2^ = 0.109), top left (F = 2.021, p = 0.147, η^2^ = 0.098) and top right (F = 0.546, p = 0.584, η^2^ = 0.029). The number of measurements had a significant simple effect on conventional training: front (F = 4.498, p = 0.018, η^2^ = 0.195), bottom left (F = 0.677, p = 0.514, η^2^ = 0.035). However, the simple effect of the number of measurements was not significant for top (F = 1.351, p = 0.281, η^2^ = 0.066), bottom (F = 0.574, p = 0.568, η^2^ = 0.030), top left (F = 1.361, p = 0.269, η^2^ = 0.069), top right (F = 0.498, p = 0.612, η^2^ = 0.026), and bottom right (F = 1.489, p = 0.239, η^2^ = 0.074).

Multiple comparisons revealed significant differences in fixation duration during the psychological procedure of free throw training across various areas: front (post-test and pre-test, mid-test; p < 0.001); top (post-test and pre-test, mid-test; p < 0.001); bottom (post-test and pre-test, mid-test; p = 0.047, p = 0.042); bottom left (post-test and pre-test, mid- test; p < 0.001). No significant differences were observed in other areas (p > 0.05). In the conventional free throw training, there were no statistically significant differences observed in fixation duration across all regions before, during, and after testing (p > 0.005).

### Pupil dilation

After S-W test, it was observed that the data from each group exhibited a normal distribution. The results of the Molloy sphericity test indicated non-conformity to the required level of sphericity for inspection criteria according to Moche’s standards of assessing conformity to spherical shape requirements in this study design (Machly W = 0.018, p < 0.001). Therefore, corrected results using one-way ANOVA with Greenhouse-Geisser adjustment will be employed.

The results of the repeated measures analysis of variance presented in Table 3 indicate that: The main effect of group did not reach statistical significance (F = 4.089, p = 0.050, η^2^ = 0.097). However, a significant main effect was observed for the number of measurements (F = 13.093, p = 0.010, η^2^ = 0.256). Furthermore, no significant interaction effect between measuring frequency and group was found (F = 2.053, p = 0.160, η^2^ = 0.051).

**Table 3:** Comparison of pupil dilation between experimental group and control group (unit: Mm) (unit: Mm).

	pre-test	mid-test	post-test	*F*	*P*	*η*2
	M±SD	M±SD	M±SD			
Experimental group	1 309±47	1 308±38	1 385±90			
Control group	1 300±39	1 001±41	1 334±61			
Group main effect				4.089	0.050	0.097
Number of tests main effect				13.093	0.001	0.256
Group * Number of tests				2.053	0.159	0.051

The results of the group simple effect test indicated that: In the pre-test, the simple effect of group was not statistically significant (F = 0.408, p = 0.527, η^2^ = 0.011). During the mid-test, there was no significant difference in the simple effect of group (F = 0.229, p = 0.635, η^2^ = 0.006). However, the post-test, a significant difference in the simple effect of group emerged (F = 4.425, p = 0.042, η^2^ = 0.104).

The results of the simple effect test on the number of measurements revealed that: In the psychological procedure of free throw training, there was a significant simple effect observed (F = 6.313, p = 0.004, η^2^ = 0.254). Conversely, for conventional training, no significant simple effect was found in relation to the number of measurements (F = 1.407, p = 0.258, η^2^ = 0.071).

The results of multiple comparisons revealed that the psychological procedure of free throw training led to a significant increase in post-test pupil dilation compared to both pre-test and mid-test pupil dilation (p = 0.001, p = 0.001). However, there was no statistically significant difference observed between pre-test and mid-test pupil diameter (p = 0.618). In contrast, conventional training did not yield any significant differences in pupil dilation among the pre-test, mid-test, and post-test conditions (p > 0.05).

### Free throw hit rate

After S-W test, it was observed that the data from each group exhibited a normal distribution. The results of the Molloy sphericity test indicated non-conformity to the required level of sphericity for inspection criteria according to Moche’s standards of assessing conformity to spherical shape requirements in this study design (Machly W = 0.139, p < 0.001). Therefore, corrected results using one-way ANOVA with Greenhouse-Geisser adjustment will be employed.

The repeated measures analysis of variance in Table 4 indicated that the main effect of group was not statistically significant (F = 0.026, p = 0.873, partial η^2^ = 0.001). Moreover, a significant main effect of the number of measurements was observed (F = 41.920, p < 0.001, η^2^ = 0.525). Additionally, there was a significant interaction effect between testing times and group (F = 13.123, p = 0.001, η^2^ = 0.257).

**Table 4:** Comparison of average free throw hit rate between experimental group and control group.

	pre-test	mid-test	post-test	*F*	*P*	*η* ^2^
	M±SD %	M±SD %	M±SD %			
experimental group	77.8±6.1	78.3±5.6	84.3±4.3			
control group	79.1±6.0	79.4±5.9	81.0±5.4			
group main effect				0.026	0.873	0.001
number of tests main effect				41.920	< 0.001	0.525
group * number of tests				13.123	0.001	0.257

The results of the group simple effect tests revealed that: In the pre-test, there was no statistically significant effect of group (F = 0.460, p = 0.502, η^2^ = 0.012). Similarly, in the mid-test, the impact of groups was also not found to be statistically significant (F = 0.401, p = 0.531, η^2^ = 0.010). However, during the post-test phase, a significant effect of group emerged (F = 4.521, p = 0.040, η^2^ = 0.106).

The results of the simple effect test on the number of measurements revealed that for the psychological procedure of free throw training, there was a significant simple effect observed (F = 26.061, p < 0.001, η^2^ = 0.585). Conversely, for conventional training, no significant simple effect was found in relation to the number of measurements (F = 2.678, p = 0.082, η^2^ = 0.126).

The results of multiple comparisons revealed that the psychological intervention involving free throw training significantly improved the post-test free throw hit rate compared to both the pre-test and mid-test (p < 0.001). Additionally, the mid-test showed a significant improvement in comparison to the pre-test (p = 0.027). In the conventional training group, the free throw hit rate in the post-test was higher compared to both pre-test and mid-test; however, this difference did not reach statistical significance (P > 0.05).

### Spearman correlation between number of fixations and free throw hit rate

Spearman’s correlation coefficient between the number of fixations and the free throw hit rate was calculated. The size of each correlation coefficient used the following evaluation criteria: < 0.1 = trivial; 0.1–0.3 = small; 0.3–0.5 = moderate; and 0.5–0.7 = large [24]. As can be seen from Table 5, there was no correlation between the number of fixations of front and the free throw hit rate (r = 0.074, P = 0.652), and there was a significant positive correlation between the number of fixations of top and the free throw hit rate, which was statistically significant (r = 0.407, p < 0.01), there was a small negative correlation between the number of fixations of bottom and the free throw hit rate (r = -0.226, P = 0.160), and there was a small negative correlation between the number of fixations of top left and the free throw hit rate (r = -0.207, P = 0.199), there was a small correlation between the duration of fixations of bottom left and the free throw hit rate (r = -0.159, P = 0.328), there was a small negative correlation between the number of fixations of top right and the free throw hit rate (r = -0.180, P = 0.266), there was no correlation between the number of fixations of bottom right and the free throw hit rate (r = -0.060, P = 0.700).

**Table 5:** Relationship between number of fixations and free throw hit rate.

		Front	Top	Bottom	Top left	Bottom left	Top right	Bottom right
Hit rate	Spearman Correlation Sig, (2-tailed) N	0.074 0.652 40	0.407** 0.009 40	-0.226 0.160 40	-0.207 0.199 40	-0.159 0.328 40	-0.180 0.266 40	-0.060 0.700 40

Notes.

**Correlation is significant at the 0.01 level (2-tailed).

### Spearman correlation between fixation duration and free throw hit rate

As can be seen from Table 6, there was a significant positive correlation between the fixation duration of front and the free throw hit rate, which was statistically significant (r = 0.573, *P* < 0.01), there was a small positive correlation between the fixation duration of top and the free throw hit rate (r = 0.267, P = 0.096) there was a small negative correlation between the fixation duration of bottom and the free throw hit rate (r = - 0.106, P = 0.517) there was no correlation between the fixation duration of top left and the free throw hit rate (r = 0.001, P = 0.996), there was no correlation between the fixation duration of bottom left and the free throw hit rate (r = -0.009, P = 0.955), there was a small negative correlation between the fixation duration of top right and the free throw hit rate (r = -0.244, P = 0.192), there was no correlation between the fixation duration of bottom right and the free throw hit rate (r = -0.004, P = 0.979).

**Table 6:** Relationship fixation duration and free throw hit rate.

		Front	Top	Bottom	Top left	Bottom left	Top right	Bottom right
Hit rate	Spearman Correlation Sig (2-tailed) N	0.573** 0.000 40	0.267 0.096 40	-0.106 0.517 40	0.001 0.996 40	-0.009 0.955 40	-0.244 0.192 40	-0.004 0.979 40

Notes.

**. Correlation is significant at the 0.01 level (2-tailed).

## Discussion

### Spatial indices of eye movements

Visual search is a complex cognitive process. When the brain obtains external visual information, the eye fixation data index reflects the visual search strategy of different cognitive tasks to a certain extent, so as to further reflect people’s psychological activities [25]. After 8 weeks of free throw psychological procedure training, the number of fixations in the experimental group has changed in different AOI. The reason is that the psychological procedure training has changed the attention range and direction of the participants during the free throw. The fixation point is concentrated near the front top, which shows concentration and directness. Reflecting the improvement of information search strategy in the experimental group.

The target fixation strategy can be explained by the visual index theory, which holds that the human visual system can distinguish the stimuli in the visual field into independent individuals based on some basic automatic operations, and this index can maintain the consistency with the object with the change of the environment [26]. The improvement of target fixation strategy in the experimental group is due to the better ability to encode and process specific information and predict the final landing of the ball after the 8-week free throw psychological procedure training. These advantages are based on the rich cognitive information database [27]. Based on the above theory, the 8-week free throw psychological procedure training the experimental group improved the attention direction, concentration and automatic computing ability of the visual system, which was specifically manifested as the high efficiency of visual search information strategy when making free throw.

### Duration index of eye movement

A longer fixation duration at a certain point during the free throw indicates a more stable fixation and a more refined information processing. After the free throw psychological procedure training, the average fixation duration in the experimental group was longer than that in the control group, indicating that the experimental group invested more time in fine processing the information in the area than the control group. According to the rules of basketball, the referee shall make a free throw within 5 seconds after placing the ball behind the free throw player [28]. The team members should complete the information search, target location, information processing, action procedure and psychological procedure within 5 seconds from receiving the ball to releasing the ball. It is necessary to optimize the fixation strategy and information processing strategy through scientific training to improve the ability of fast searching for the target in a short time and the ability of fine processing the information in the fixation area. It is impossible to complete the search and location of all targets and perform fine information processing and analysis in a limited time. It can only optimize the fixation target, focus on the fixation position that has a greater impact on the free throw shooting rate, and extract useful information for fine processing. The 8-week free throw psychological procedure training also plays a role in optimizing the information search and processing strategy. This could also explain the unstable fixation and blind information search during free throws in the control group. This study is consistent with the views of previous researchers, and the players with higher shooting percentages fixation longer [29]. In physical education teaching and training, attention training is helpful to improve sports performance [30]. Players with higher shooting longer fixation duration.

### Pupil dilation

When people process information, pupil dilation will change, and the magnitude of the change is closely related to the degree of mental effort during information processing. This index mainly reflects the degree of cognitive processing and the size of cognitive processing load [31]. The higher the intensity of cognitive processing, the larger the pupil diameter of the subject [32], the more interested in the area of fixation during the free throw, and the larger the pupil dilation during information processing. The pupil dilation of the experimental group increased after the free throw psychological procedure training. The reason may be that the free throw psychological procedure training makes it easier for the participants to focus their attention on the key movement information. Every penalty basket has a clear fixation target position, the attention is highly concentrated, the mental load increases, and a stable fixation pattern is formed in the long-term practice. Every free throw is highly serious and actively focused on the target area; The control group had unstable fixation during the free throw, and their vision was always in search during the free throw, so they could not focus their attention on the area that had important influence on the free throw, and their cognitive processing load was small, so the pupil dilation was not as large as that of the experimental group.

### Analysis of number of fixations, fixation duration and free throw hit rate

The ability of visual searching and processing information in the process of free throw has an important impact on the hit rate of mobilizing free throw. Before players make action responses, useful visual information must be selected and analyzed [33]. In order to improve and maintain a high free throw hit rate, it is essential to maintain a high degree of attention and a stable fixation position. Psychologically, the key is to focus on the proper range when shooting [23]. No matter what kind of sports, coaches and players all believe that attention is the key to directly affect the competitive level and win the competition. Since the attention mode is closely related to the competition task and affects the activities and competitive performance of athletes, it is essential to cultivate and improve the attention ability of players [34].

After 8 weeks of free throw psychological procedure training, the average free throw hit rate in the experimental group was significantly increased, indicating that the free throw psychological procedure training improved the information search strategy and information fine processing ability of the participants, and constructed a stable attention mode, which significantly improved the free throw hit rate. From the number of fixations and free throw hit rate, it can be found that at top, the number of fixations and free throw hit rate showed a significant positive correlation, and the relationship between fixation duration and free throw hit rate showed that at front, the fixation duration and free throw hit rate was significantly positive correlation. These evidences indicate that to maintain a high free throw hit rate, players should look at front and top when free throw. At front, the fixation duration should be long enough. This study is consistent with the previous research conclusion that athletes with high shooting accuracy have a longer fixation time [35–37].

The reason may be that more time is consumed in other AOI to shorten the information processing time of the key area, finally, due to the time pressure, the shot can only be rushed, and the free throw hit rate is reduced. Ripoll, et al. believed that the change of visual attention point in the shooting process would affect the stability of the shooting, thus affecting the shooting effect [38]. Steciuk et al. also believed that longer target fixation duration and fewer number of fixations were conducive to more accurate shooting [39]. The above evidence provides a scientific basis for sports teaching and training. Basketball shooting teaching and training should guide students or players to focus on front and top, which is helpful to improve or maintain higher shooting hit rate.

## Conclusion

The characteristics of visual attention of the participants were changed when the free throw psychological procedure training applied to the free throw, the information search strategy and information processing ability of the fixation target were improved, and the new fixation mode is constructed and the free throw hit rate is significantly improved. There was a positive correlation between the front fixation time and the free throw hit rate, and there was a positive correlation between the top number of fixations and the free throw hit rate.

## References

[1] Xu H W. Basketball team management and psychological training. China Intellectual Property Press; 2012.

[2] Hong XB, LIU XR, LI MH. An Experimental Study of the Influence of Pre-performance Routines on the Percentage of Hits of Basketball Free Throw. J Capital Institute of Physical Education. 2007; 19(2): 49–50. ISSN: 1009-783X.

[3] Nideffer RN. Concentration and attention control raining. In J. M. Williams (Ed.), Applied sp ort psychology: Personal growth to peak performance (2nded). Mountain View, CA: Mayfield. 1993; 243–261.

[4] Vickers J. Control of Visual Attention during the Basketball Free Throw. Am J Sports Med 24(6Suppl).1996; S93–7. . 8947439

[5] Netolitzchi M, Leonte N, Popescu O, Branet C. Increasing precision and optimising efficiency in distance basketball shooting (6.75 m). Discobolul–Physical Education, Sport and Kinetotherapy Journal. 2019; 25:160–164 doi: 10.35189/iphm.icpesk.2019.25 ISSN 2668-9405.

[6] Jiang X. A preliminary study on the relationship between spatial stereo vision of basketball athletes and shooting accuracy. J Songyuan: Sci Nat Ed.2000; (1): 81–82. doi: 10.16862/j.cnki.issn1674-3873.2000.01.030.ISS:1674-3873

[7] Liang NJ. Contemporary Cognitive Psychology. Shanghai Educational Publishing House.2014: 99–100.

[8] Mizuguchi N, Honda M, Kanosue K. Prediction of shot success for basketball free throws: visual search strategy. Eur J Sport Sci. 2013; 14(5):426–432. doi: 10.1080/17461391.2013.866166 . 24319995

[9] Erickson KI, Voss MW, Prakash PS, et al. Exercise training increases size of hippocampus and improves memory. Proc Natl Acad Sci U S A. 2011, 108(7):3017–3022. doi: 10.1073/pnas.1015950108 . 21282661 PMC3041121

[10] Yanagisawa H, Dan I, Tsuzuki D, et al. Acute moderate exercise elicits increased dorsolateral prefrontal activation and improves cognitive performance with Stroop test. Neuroimage. 2010; 50(4): 1702–1710. doi: 10.1016/j.neuroimage.2009.12.023 . 20006719

[11] Vickers JN, Ben V, Christie K, Brendan R.Quiet eye training improves accuracy in basketball field goal shooting. Prog Brain Res. 2017; 234: 1–12. doi: 10.1016/bs.pbr.2017.06.011 . 29031458

[12] Du Q, Wu ZH. The effect of aiming point and proprioception on the shooting percentage of basketball players. Abstracts of the 11th Chinese National Sports Science Congress. 2019; 11: 4612–4614. doi: 10.26914/c.cnkihy.2019.030945

[13] Jiméne Z, Sánchez AC, Sáenz LP, Lorenzo CA, et al. Decision making of spanish female basketball team players while they are competing. Revista de Psicologia del Deporte. 2009; 18(3): 369–373. ISSN: 1132-239X.

[14] Chuang L Y, Huang C J, Huang TM. The differences in frontal midline theta power Between successful and unsuccessful basketball free throws of elite basketball players. Int J Psychophysiol, 2013, 90(3): 321. doi: 10.1016/j.ijpsycho.2013.10.002 . 24126125

[15] Mangine GT, Hoffman J R, Wells A J, et al. Visual tracking speed is related to basket-ball specific measures of performance in NBA players. J Strength Cond Res. 2014; 28(9): 2406. doi: 10.1519/JSC.0000000000000550 . 24875429

[16] Harle SK, Vickers JN. Training quiet eye improves accuracy in the basketball free throw. Sport Psychol. 2001; 15(3):289–305. doi: 10.1123/tsp.15.3.289 ISSN: 0888-4781.

[17] Daly GD, Bornn. Rao-Blackwellizing field goal percentage. Journal of Quantitative Analysis in Sports.2019; 15(2):85–95.

[18] Chen B, Ren J. The mechanical analysis of shooting aiming point. Journal of Taiyuan Unive- rsity of Technology. 2006; 37(1):29–30. ISSN 1007-9432.

[19] Qiu F, Pi Y, Liu K, Zhu H, Li X, Zhang J, Wu Y. Neural efficiency in basketball players is associated with bidirectional reductions in cortical activation and deactivation during multiple-object tracking task performance. Biol Psychol. 2019; 144:28–36 doi: 10.1016/j.biopsycho.2019.03.008 2019.03.008. . 30902565

[20] Sagi D. Perceptual learning in Vision Research. Vision Research. 2011; 51(13): 1552–1566. doi: 10.1016/j.visres.2010.10.019 ISSN: 0042-6989. 20974167

[21] Cohen J. Statistical power analysis. Curr Dir Psychol Sci.1992;1(3):98–101. 10.1111/1467-8721.ep10768783 ISSN:0963-7214.

[22] Lakens D. Performing high-powered studies efficiently with sequential analyses. Eur J Soc Psychol. 2014; 44(7): 701–710. doi: 10.1002/ejsp.2023 ISSN:0046-2772.

[23] Kevin L B, Dale B. Sport Psychology: Basketball (Zhang ZQ., et al., Trans). Mechanical Eng- ineering Press; 2005: 52.

[24] Hopkins WG, Marshall SW, Batterham AM, Hanin J. Progressive statistics for studies in sports medicineand exercise science. Med Sci Sports Exerc.2009;41(1): 3–13. doi: 10.1249/MSS.0b013e31818cb278 . 19092709

[25] Jin P. The visual attention characteristics of basketball players and its relationship to match performance. (Education PhD Thesis), Shanghai University of Sport. 2020.

[26] Goss S, Hall CR, Buckolz E, Graham F. Imagery ability and the acquisition and retention of movements. Memory & Cognition. 1986; 14:469–477. doi: 10.3758/bf03202518 ISSN: 0090 -502X. 3796284

[27] Williamss AM, Davids K, Burwitz L, Williams JG. Visual search strategies in experienced and inexperienced soccer players. Res Q Exe Sport. 1994; 65(2):127–135. doi: 10.1080/02701367 . 8047704

[28] Chinese B A. Rules of basketball. Beijing Sport University Press, 2019: 117.

[29] Zhu Y, Gao J, Huang B, et al. Analysis of the Thinking Control Characteristics of Basketball Free Throws Based on Eye Movement and EEG.J Tianjin Univ. Sport. 2014; 29(4):313–318. DOI:10.13297/j.cnki.issn1005-0000. 2014.04.008. ISSN: 1005-0000.

[30] Gou QF, Li SN. Study on the correlation between basketball players’ multiple-object tracking ability and sports decision-making. PLOS One. 2023; 18(4): 1–12. doi: 10.1371/journal.pone.0283965 . 37018189 PMC10075393

[31] Robin CJ, Peter M. Advance Visual Information, Awareness, and Aniticipation Skill. J Mot Behav. 2007; 39(5): 341–351. doi: 10.3200/JMBR.39.5.341-352 . 17827112

[32] Zhang X M, Liao Y G, Ge C L. Study on Eye Movement Characteristics of Volleyball Player in Sport Scene. China Sport Science. 2008; 6(28):57–61. doi: 10.3969/j.issn.1000-677X 2008. 06.009. ISSN: 1000-677X.

[33] Xi J, Wang Q, Yan G. Eye Movement Analysis and Sports Psychology Research. Stud Psychol Behav. 2004; 2(3): 555–560. https://psybeh.tjnu.edu.cn/CN/. ISSN: 1672-0628.

[34] Mao Z X, Chi L Z. Psychology of sports. China Renmin University Press, 2021: 53.

[35] Vrickers J N. Visual Control When Aiming at a Far Target. J Exp Psychol Hum Percept Perfo- rm. 1996; 22(2): 324–354. doi: 10.1037//0096-1523.22.2.342 . 8934848

[36] Harle SK, Vickers JN. Training quiet eye improves accuracy in the basketball free Throw. Sport Psychol. 2001; 15(3):289–305. doi: 10.1123/tsp.15.3.289 ISSN: 1543-2793.

[37] Vinw SJ, Moore L J, Wilson MR. Quiet eye training: the acquisition, refinement and resilient performance of targeting skills. Eur J Sport Sci. 2014; 14(1): 235–242. doi: 10.1080/17461391.2012.683815 . 24444212

[38] Ripoll H, Bard C, Paillard J. Stabilization of head and eyes on target as a factor in successful basketball shooting. Hum Mov Sci. 1986; 1(5):47–58. doi: 10.1016/0167-9457(86)90005-9 ISSN: 18727476.

[39] Steciuk H, Zwierko T. Gaze behavior in basketball shooting: Preliminary investigateons. Tre- nds Sport Sci. 2015. 22(2): 89–94. Gaze behavior in basketball shooting: Preliminary investigations (sponet.fi). ISSN: 2299-9590.

